# Improved detection and management of advanced HIV disease through a community adult TB‐contact tracing intervention with same‐day provision of the WHO‐recommended package of care including ART initiation in a rural district of Mozambique

**DOI:** 10.1002/jia2.25775

**Published:** 2021-08-04

**Authors:** Santiago Izco, Adrià Murias‐Closas, Alexander M Jordan, Gregory Greene, Nteruma Catorze, Helio Chiconela, Juan Ignacio Garcia, Alejandro Blanco‐Arevalo, Anna Febrer, Aina Casellas, Belén Saavedra, Tom Chiller, Tacilta Nhampossa, Alberto Garcia‐Basteiro, Emilio Letang

**Affiliations:** ^1^ ISGlobal, Hospital Clínic‐Universitat de Barcelona Barcelona Spain; ^2^ Centro de Investigação em Saude de Manhiça (CISM) Manhiça Mozambique; ^3^ Mycotic Diseases Branch United States Centers for Disease Control and Prevention (CDC) Atlanta GA USA; ^4^ National Tuberculosis Program Manhiça Mozambique; ^5^ PhD Program in Methodology of Biomedical Research Faculty of Medicine University of Barcelona Barcelona Spain; ^6^ Internal Medicine Department Hospital Universitari de Bellvitge Barcelona Spain; ^7^ Department of Infectious Diseases Hospital del Mar Hospital del Mar Research Institute (IMIM) Barcelona Spain

**Keywords:** AHD, TB contact‐tracing, CrAg, TB‐LAM, ART initiation, sub‐Saharan Africa

## Abstract

**Introduction:**

AIDS‐mortality remains unacceptably high in sub‐Saharan Africa, largely driven by advanced HIV disease (AHD). We nested a study in an existing tuberculosis (TB) contact‐tracing intervention (Xpatial‐TB). The aim was to assess the burden of AHD among high‐risk people living with HIV (PLHIV) identified and to evaluate the provision of the WHO‐recommended package of care to this population.

**Methods:**

All PLHIV ≥14 years old identified between June and December 2018 in Manhiça District by Xpatial‐TB were offered to participate in the study if ART naïve or had suboptimal ART adherence. Consenting individuals were screened for AHD. Patients with AHD (CD4 < 200 cells/μL or WHO stage 3 or 4) were offered a package of interventions in a single visit, including testing for cryptococcal antigen (CrAg) and TB‐lipoarabinomannan (TB‐LAM), prophylaxis and treatment for opportunistic infections, adherence support or accelerated ART initiation. We collected information on follow‐up visits carried out under routine programmatic conditions for six months.

**Results:**

A total of 2881 adults were identified in the Xpatial TB‐contact intervention. Overall, 23% (673/2881) were HIV positive, including 351 TB index (64.2%) and 322 TB contacts (13.8%). Overall, 159/673 PLHIV (24%) were ART naïve or had suboptimal ART adherence, of whom 155 (97%, 124 TB index and 31 TB‐contacts) consented to the study and were screened for AHD. Seventy percent of TB index‐patients (87/124) and 16% of TB contacts (5/31) had CD4 < 200 cells/µL. Four (13%) of the TB contacts had TB, giving an overall AHD prevalence among TB contacts of 29% (9/31). Serum‐CrAg was positive in 4.6% (4/87) of TB‐index patients and in zero TB contacts. All ART naïve TB contacts without TB initiated ART within 48 hours of HIV diagnosis. Among TB cases, ART timing was tailored to the presence of TB and cryptococcosis. Six‐month mortality was 21% among TB‐index cases and zero in TB contacts.

**Conclusions:**

A TB contact‐tracing outreach intervention identified undiagnosed HIV and AHD in TB patients and their contacts, undiagnosed cryptococcosis among TB patients, and resulted in an adequate provision of the WHO‐recommended package of care in this rural Mozambican population. Same‐day and accelerated ART initiation was feasible and safe in this population including among those with AHD.

## 1 Introduction

Despite the reduction in HIV incidence and AIDS‐mortality resulting from expanded access to antiretroviral treatment (ART) globally, more than one‐third of people living with HIV (PLHIV) continue to present to care with advanced HIV disease (AHD) in many low and middle income countries [1, 2, 3]. AHD is defined as having <200 CD4 cells/μL or World Health Organization (WHO) clinical stage 3 or 4 criteria [4]. People presenting with AHD are at high risk of opportunistic infections (OI), hospitalizations and mortality with tuberculosis (TB) and cryptococcosis accounting for the majority of these AIDS‐related deaths [5, 6, 7, 8, 9, 10, 11, 12].

In 2015, the WHO called for differentiated care service‐delivery for various HIV populations [6, 13, 14, 15]. Two years later specific guidelines for managing AHD were released [4]. These promote offering PLHIV a “package of interventions” including rapid OI screening, prompt OI prophylaxis/treatment and accelerated ART initiation. They include a decision‐making guide to assist clinicians in rapid implementation of these measures, since the need for repeated visits has proved to increase pre‐ART attrition [16, 17, 18]. In this sense, point‐of‐care (POC) diagnostics are recommended to allow for patient‐centred services in a single‐visit even in remote healthcare settings [16, 19, 20]. Although there is a growing body of evidence supporting the benefit of these interventions, their translation into practice remains limited [14, 15, 21, 22].

In January 2018, the *Centro de Investigação em Saúde de Manhiça* (CISM; Manhiça Health Research Centre), in collaboration with the National Tuberculosis Program (NTP) officers in the Manhiça District of Southern Mozambique began implementing a project named Xpatial‐TB. This project assessed a novel community‐based, TB active case‐finding (TB‐ACF) strategy among risk‐stratified adult and child contacts of all newly diagnosed TB cases registered in the district.

Ancillary to the Xpatial‐TB study, we nested a study with two objectives: (i) assessing the burden of AHD among high‐risk HIV‐positive adults identified through Xpatial‐TB; (ii) evaluating the implementation of the WHO‐recommended package of care among PLHIV with AHD [4].

## 2 Methods

### 2.1 Xpatial‐TB (TB‐contact tracing) intervention

In 2018, incident TB cases (either pulmonary or extrapulmonary) registered in any TB diagnostic unit in the district and their household and community contacts of any age were visited. These were defined as population living within radios of 40, 70 or 120 m from the index case, depending on the cycle threshold (Ct) values of their Xpert MTB/RIF Ultra (Ultra^®^) result and population density. Tracing was done using the information provided by the existing Health and Demographic Surveillance System (HDSS), which covers the whole Manhiça district. Xpatial‐TB field workers interviewed TB cases and contacts about their HIV‐status, offered HIV counselling and testing (HCT). In addition, among those contacts HIV positive or with a positive WHO‐four symptom screening (WHO‐4SS, any duration of cough, fever, night sweats or weight loss), a sputum sample was obtained through a portable nebulizer as needed. All contacts ≤14 years old were booked at the Xpatial‐TB study clinic for clinical evaluation including further sampling (nasopharyngeal and/or gastric aspirate, stool) and a chest X‐ray for those ≤5 years old. Adult contacts newly diagnosed with HIV positive and/or TB positive were given a referral letter to the health system.

### 2.2 Study intervention

From 28 May to 31 December 2018, consenting PLHIV ≥ 14 years old reached by Xpatial‐TB were offered to participate in this ancillary study if they had been newly diagnosed with HIV, were ART interrupters or had poor ART adherence regardless of the Xpatial‐TB status (TB index or contacts). Upon acceptance, a study counsellor visited the consenting participants within 24 hours and offered them a home‐counselling session followed by transport to the study clinic, where a clinical officer provided: (i) Clinical evaluation, including the assessment of danger signs (heart rate >120 beats per minute, respiratory rate >30 breaths per minute, systolic blood pressure <90 mmHg, body temperature >39° Celsius, moderate or severe dehydration, bedridden or needing aid to walk, altered mental state, other neurological problem); (ii) WHO HIV clinical staging; (iii) venous sampling for CD4 cell counts (BD FACS Calibur, BD Biosciences, Franklin Lakes, NJ, USA) and baseline pre‐ART safety liver and kidney biochemistry analysis; (iv) a reflex cryptococcal antigen lateral flow assay (CrAg‐LFA, Immuno‐Mycologics, Inc., OK, USA) that was performed in the laboratory if <200 CD4 cells/μL; (v) a urine tuberculosis lateral flow lipoarabinomannan test (TB‐LAM, Alere Inc., Waltham, MA, USA) done if any danger sign was present or <200 CD4 cells/μL and (vi) a digital chest X‐Ray (CXR). Of note, CrAg‐screening and TB‐LAM were not routine of care in the district at the time. The latter was performed by the study officer in the clinic regardless of the TB status of the participants.

**Figure 1:**
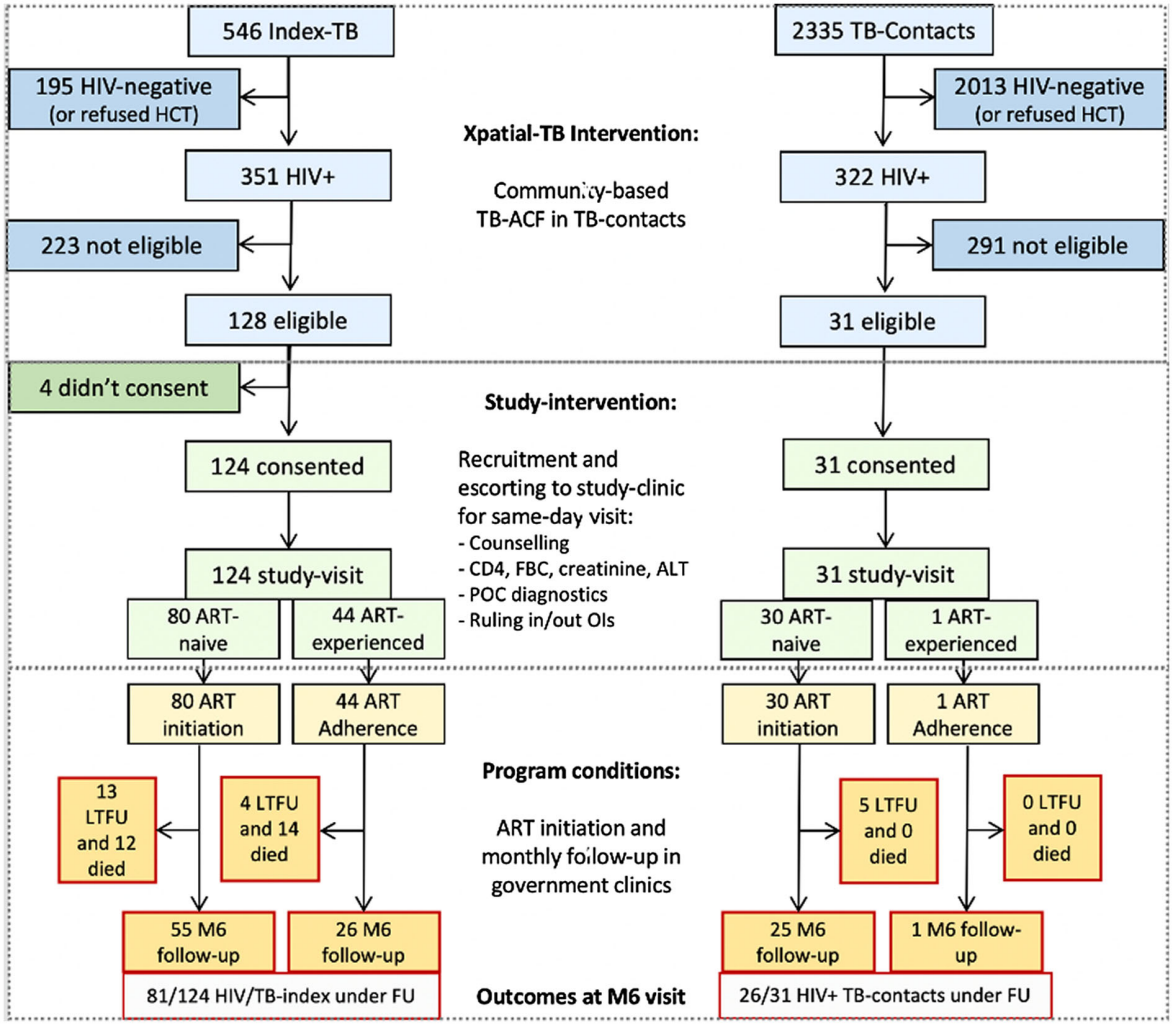
Flow chart of interventions. Blue boxes, Xpatial‐TB interventions. Green boxes, study‐intervention. Yellow boxes, routine provision of care in district ART units (government health centers). ALT, alanine‐amino‐transferase; ART, antiretroviral therapy; FBC, full blood count; FU, follow‐up; HCT, HIV counselling and testing; LTFU, lost to follow‐up; M6, month six; OI, opportunistic Infections; POC, point of care; TB‐ACF, tuberculosis active case finding.

Following positive serum CrAg results, lumbar puncture (LP) was done for CrAg testing of cerebrospinal fluid (CSF). The clinician prescribed treatment for OIs including TB and cryptococcal meningitis (CM), isoniazid preventive therapy (IPT) for TB contacts after ruling out TB, cotrimoxazole preventive therapy (CPT) for TB cases regardless of CD4 count and contacts with CD4 < 350 cells/μL and fluconazole pre‐emptive therapy for patients with a positive serum CrAg but no CM. All these interventions were performed at the same single visit. After completing them, participants met again with the study counsellor to discuss readiness for ART initiation. Data collection of this single study visit was captured by the clinical officer and counsellors with tablets using OpenDataKit open‐source data‐capture software (Figure 1).

### 2.3 ART initiation or ART adherence enforcement

On the same day or on the following day for those from most remote locations, the study counsellor accompanied the patient to their public HIV clinic. There, a report of the study visit was delivered to the corresponding public officer for them to make a decision on timing for OI treatment and ART initiation. At this point, the role of the study counsellor was limited to document patient registration, prescriptions and patient picking‐up of medication.

Participants included due to suboptimal ART adherence were also escorted to their health units, where they were booked for the routine government programme of adherence counselling and plasma HIV viral load (VL) monitoring as recommended by the National ART guidelines [23, 24].

### 2.4 Follow‐up visits

Participants were followed‐up at their respective government health units under routine programmatic conditions. The study was limited to retrieve information from these clinics on visit attendance and prescription refills of the participants. In case a visit record was unavailable, the study counsellor made a maximum of three attempts to trace the participant and advise them to resume their clinic visits (Figure 1).

### 2.5 Outcome definitions and mortality

Information on mortality was gathered from the existing HDSS and death registries at each health unit. Loss‐to‐follow‐up (LTFU) was defined as failure to attend the six‐month visit within a 30‐day window. Attrition was defined as LTFU or death before the sixth scheduled visit.

### 2.6 Statistical analysis

Data were described by frequencies and median (interquartile range, IQR) for discrete and continuous variables, respectively. Cox regression was used to assess the effect of potential risk factors on the probability of TB‐index cases being retained in care until the sixth month after enrolment. A multivariable Cox proportional hazards model was obtained using a stepwise procedure, starting with a model including variables with clinical relevance defined from literature review (age, sex, severe anaemia, CrAg positivity, TB‐LAM positivity and low CD4 count). These variables were kept in the model. Entry and removal criteria for the remaining variables were based on the *p*‐value of the univariate analysis (<0.05 and >0.10 respectively). The statistical significance level was set at 0.05. All statistical analyses were conducted using Stata (StataCorp. 2019;StataCorp LLC., College Station, TX, USA).

## 3 Results

Between 28 May and 31 December 2018, 2881 adults were visited in the Xpatial‐TB intervention, including 546 TB‐index cases and 2335 of their contacts. Of those, 351 (64%) and 322 (14%) were HIV positive, respectively. Overall, eligibility criteria for our ancillary intervention (newly HIV diagnosed, ART naïve, suboptimal ART adherence and ART interrupters) were met by 128 TB‐index cases and 31 contacts. Of those, 98% (124/128) TB‐index cases and all 31 contacts agreed to enrolment (Figure 1). Of the TB‐index cases, 86% (107/124) were pulmonary TB (PTB), of whom 36% (38/107) had a positive Ultra^®^ result and 52% (65/124) were hospitalized at the time of enrolment. All 31 contacts were ambulatory (Table 1).

**Table 1:** Characteristics of the study population

Variable	Participant type		Total (n = 155)
	TB‐index cases (n = 124)	TB contacts (n = 31)	
Age, years	36.0 (30.6 to 44.5)	34.0 (27.0 to 39.7)	35.9 (29.9 to 43.8)
Male sex	72 (58)	11 (35)	83 (54)
Inpatient at recruitment	65 (52)	0	65 (42)
Body mass index^a^, kg/m^2^	19.4 (17.5 to 21.5)	23.4 (20.8 to 25.6)	19.9 (17.9 to 23.0)
Haemoglobin < 8 g/dL	29 (23)	1 (3)	30 (19)
Abnormal ALT (≥1.5 ULN)	10 (8)	0	10 (6)
eGFR<60 mL/min/1.73 m^2^	20 (16)	0	20 (13)
CD4 cells/μL	105.0 (38.5 to 258.5)	355.0 (255.0 to 605.0)	132.0 (47.0 to 337.0)
Danger signs	75 (60)	2 (6)	77 (50)
ART status			
New case	28 (23)	16 (52)	44 (28)
ART naïve	36 (29)	5 (16)	41 (26)
Suboptimal adherence	44 (35)	1 (3)	45 (29)
Interrupter	16 (13)	9 (29)	25 (16)
WHO stage			
Stage 1 or 2	0	26 (84)	26 (17)
Stage 3 or 4	124 (100)	5 (16)	129 (83)
CD4 < 100 cells/μL	60 (48%)	1 (3%)	61 (39%)
CD4 < 200 cells/μL^b^	87 (70)	5 (16)	92 (59)
Advanced HIV disease^c^	124 (100)	9 (29)	133 (86)

Data are shown as median (IQR) or n (%). ALT, alanine aminotransferase; eGFR, estimated creatinine glomerular filtration rate GFR (CKD‐EPI formula); ART, antiretroviral therapy; Danger signs, any of the following present: Heart rate >120 beats per minute, respiratory rate >30 breaths per minute, Systolic blood pressure <90 mmHg, body temperature >39° Celsius, moderate or severe dehydration, bedridden or needing aid to walk, altered mental state, other neurological problem.

^a^ n=153 (two missing values)

^b^ includes also those with <100 cells/μL

^c^ AHD defined as having CD4 < 200 cells/μL or WHO stage 3 or 4.

### 3.1 Characteristics of the study population and prevalence of advance HIV disease

Since TB is a WHO stage 3 or 4 condition, all 124 TB‐index cases had AHD by definition. Additionally, other WHO stage 3 or 4 conditions were present in 76% (94/124) of TB index. CD4 counts were <200 cells/μL in 70% (87/124). Of the 31 contacts, five (16%, 5/31) had a WHO stage 3 or 4 condition, including four (13%, 4/31) with TB. Five contacts (16%, 5/31) had <200 CD4 cells/μL. Only one contact met both clinical and immunologic criteria. Overall, any AHD criteria were met by 29% (9/31) of TB contacts (Table 1).

### 3.2 Screening and diagnosis of tuberculosis disease and cryptococcosis

Among TB‐index cases, 78% (97/124) met TB‐LAM testing indications. Of those, 96% (93/97) were TB‐LAM tested and 70% (65/93) had a positive result. TB‐LAM was the only positive TB‐test result in 51% (33/65) and 17% (10/59) of hospitalized and ambulatory TB‐index patients, respectively. Overall, Ultra^®^ was the only positive TB result in 11% (7/65) and 31% (18/59) of hospitalized and ambulatory TB‐index cases, respectively. A CXR was done in 88% (109/124) of TB‐index participants, being interpreted as suggestive of TB in 81% (88/109).

Among contacts, 23% (7/31) had a positive WHO‐4SS. Tuberculosis was diagnosed in four cases (4/31, 13%) including one that was 4SS−. One diagnosis was obtained only by TB‐LAM (4SS+), two only by Ultra^®^ (one 4SS+, one 4SS−) and one was clinically diagnosed (4SS+) with a suggestive CXR.

Serum CrAg screening result was available for all 92 eligible participants (CD4 counts ≤200 cells/μL) and was positive in 4.9% (3/61) and 4.3% (4/92) of those with ≤100 and ≤200 CD4 cells/μL respectively. All four CrAg‐positive cases were TB‐index patients and were performed a CSF‐CrAg test that resulted negative in two of them (both were asymptomatic, including the participant with >100 CD4 cells/μL) and positive in two cases that had also disseminated TB and confirmed TB meningitis, respectively. Both had severe neurological symptoms that had been regarded as TB related prior to the serum CrAg‐positive result.

### 3.3 Prevention and treatment of opportunistic infections

IPT was started for 93% (25/27) TB‐free contacts. No new episodes of TB were reported among participants. CPT was provided to 98% (138/141) of cases with ≤350 CD4 cells/μL or TB. Of the four plasma CrAg‐positive participants, the two with a negative CSF CrAg started fluconazole pre‐emptive therapy. Both were alive and under follow‐up at the end of the six‐month follow‐up. The two TB/CM co‐infected (CSF‐CrAg+) continued antitubercular treatment and received short‐induction with amphotericin B‐deoxycholate and high dose fluconazole followed by six weeks of fluconazole consolidation, after which they started ART and fluconazole maintenance therapy. One was in good health at the end of study follow‐up, but the other died seven weeks after ART initiation. The four contacts diagnosed with TB started on TB treatment followed by timely ART initiation and were all alive at the end of the study period.

### 3.4 ART initiation

**Figure 2:**
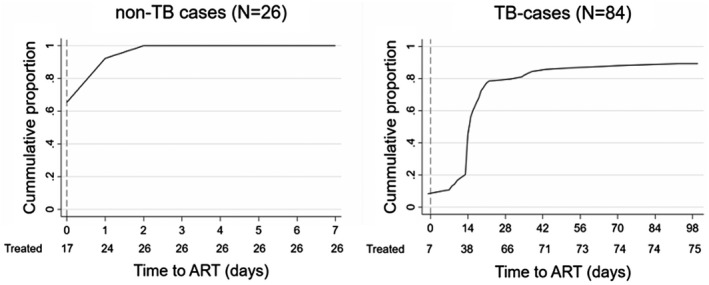
Time to ART‐initiation. Daily cumulative proportion of participants (ART‐naïve and ART interrupters) starting or resuming ART as part of the intervention (‘treated’). Left, TB‐free participants; Right, TB cases including TB‐index and TB‐contacts with TB. Four additional TB‐cases started ART 148, 148, 246 and 247 days after enrolment respectively.

All 80 TB‐index cases (80/124, 65%) and 30 contacts (30/31, 97%) that were not on ART at recruitment initiated or resumed ART in the intervention. Of these, 84 TB cases, including all 80 index cases and the four TB contacts diagnosed with TB delayed ART a median of 15 days (IQR 14 to 19) after initiation of antitubercular treatment. The two cases with TB/CM started ART at day 38 and 54 respectively. All TB‐negative contacts initiated ART within 48 hours of their recruitment (Figure 2).

### 3.5 ART adherence re‐enforcing

All 44 TB‐index cases (44/124, 35%) and one contact (1/31, 3%) included in the study because of suboptimal ART adherence were counselled and booked in their public clinics for adherence re‐enforcement counselling. None of the participants had been switched to second‐line ART by the end of the study follow‐up. Public officers in district clinics reported to the study that they had sent samples to Mozambique’s capital for VL determination as per national guidelines, but no result had been recorded in the files during the six‐month study period.

### 3.6 Retention and survival

Six‐month mortality was 21% (26/124) in TB index of which 88% (23/26) occurred during admission and 0% (0/31) in contacts. Six‐month LTFU occurred in 14% (17/124) of TB index and 16% (5/31) of contacts.

Male sex was the only independent predictor of attrition identified (adjusted Hazard Ratio, aHR 2.18, 95% CI 1.10 to 4.34) adjusted by age, severe anaemia, CrAg positivity, TB‐LAM positivity and CD4 count. A trend was observed between severe anaemia and increased hazard of attrition aHR 2.01, 95% CI 0.97 to 4.17 (Table 2).

**Table 2:** Factors associated with attrition in TB‐index participants

	Univariable analysis			Multivariable analysis		
Variable	Crude hazard ratio	(95% confidence interval)	*p*‐value	Adjusted hazard ratio	(95% confidence interval)	*p*‐value
Age^a^	0.99	(0.96 to 1.01)	0.297	0.98	(0.95 to 1.01)	0.149
Male sex	1.42	(0.77 to 2.62)	0.261	2.18	(1.10 to 4.34)	0.026
Severe anaemia (Hb <8 g/dL)	2.23	(1.20 to 4.18)	0.012	2.01	(0.97 to 4.17)	0.062
Renal insufficiency (eGFR < 60 mL/min)	1.13	(0.55 to 2.32)	0.732	–	–	
CrAg positivity	0.65	(0.15 to 2.75)	0.558	0.55	(0.12 to 2.55)	0.446
TB‐LAM positivity	1.94	(1.05 to 3.60)	0.035	1.38	(0.67 to 2.85)	0.383
Cotrimoxazole preventive therapy	0.36	(0.05 to 2.70)	0.323	–	–	–
Low CD4 (<200 cells/μL)	1.96	(0.81 to 4.74)	0.133	1.72	(0.65 to 4.52)	0.271
Suboptimal adherence versus ART initiated	1.72	(0.94 to 3.14)	0.077	–	–	

Cox regression, crude model (left) and adjusted model (right). Number of subjects = 124. eGFR, Estimated creatinine glomerular filtration rate GFR (CKD‐EPI formula).

^a^ Per unit increase.

## 4 Discussion

The aim of this study was to describe the burden of advanced HIV disease among TB‐index cases and their contacts and to assess the feasibility of implementing the WHO package of interventions for AHD in this population. Key findings of the study are as follows: (i) a high prevalence of AHD among HIV‐positive TB contacts (approximately 1/3rd); (ii) a CrAg prevalence over 4% among TB‐index cases; (iii) TB‐LAM was the only positive TB test in half of TB inpatients (iv) almost 100% initiation of IPT, CPT and fluconazole pre‐emptive treatment and 100% coverage of early ART initiation through a same‐day community intervention; (v) six‐month attrition and mortality comparable with that of historical records among TB cases and no mortality among their HIV‐positive contacts, including those with AHD.

We nested an ancillary intervention into an existing community‐reach TB‐case finding strategy (Xpatial‐TB) that was being assayed in a rural district in Mozambique. In seven months, Xpatial‐TB had reached 546 adult TB‐index cases of whom 351 (64%) were HIV co‐infected. Over one‐third of these were newly HIV diagnosed, ART interrupters or had suboptimal ART adherence (the present criteria defining our target population). We visited these patients and found a 70% prevalence of AHD based on CD4 counts. This finding aligns with previous reports of suboptimal HIV control among the TB/HIV population [25, 26, 27, 28, 29, 30, 31].

During the same timeframe, Xpatial‐TB visited 2335 adult TB contacts of whom 322 (14%) were HIV positive. Of them, 31 (10%) met the mentioned criteria, including 30 HIV‐positive contacts newly HIV diagnosed and one with suboptimal ART adherence. One‐third of these HIV‐positive TB contacts had AHD (29%, 9/31), either immunologically defined (CD4 <200 cells/μL in 5/9) and/or clinically defined (WHO stage 3 or 4 found in 4/9). The overall proportion of AHD is in line with other AHD surveys from the region [1, 2, 3]. TB programme managers should factor in AHD screening and linkage to care of HIV‐positive TB contacts when discussing the efficiency of community TB contact‐tracing interventions, which have been often regarded as low efficient when the benefit is set only in their TB diagnosis yield [32, 33, 34, 35, 36, 37, 38, 39].

CrAg prevalence was 4.3% and 4.9% in those with ≤200 and ≤100 CD4 cells/μL, respectively, similar to previous estimates of prevalence of cryptococcosis among PLHIV in sub‐Saharan Africa (SSA) [7, 8, 9, 40, 41, 42]. Of note, if we had used the 100 cells/μL threshold for screening, one out of four CrAg‐positive cases would have been missed. All CrAg‐positive cases were found among TB patients. When caring for TB/HIV patients, TB officers are facing clinically defined AHD, with a majority of these having low CD4 counts. However, CrAg‐screening, a cost‐efficient life‐saving intervention for patients with AHD, is often missed in the integration of HIV and TB services [28, 43, 44, 45, 46, 47].

There was no pre‐ART attrition in the study. Embedding the intervention within TB‐contact tracing and offering a full TB‐diagnostic work‐up even for asymptomatic HIV‐positive TB contacts may have helped acceptance of the clinic visit. HIV counsellors accompanied newly HIV‐positive diagnosed participants throughout the day‐long process leading up to the first ART pick‐up. Other studies have shown the importance of similar escorting strategies to help linkage to care [48, 49]. Finally, the same‐day full pre‐ART evaluation may have helped pre‐ART retention when compared to the standard process that entails multiple visits [16, 17, 32, 50, 51].

Ninety percent of TB cases started or resumed ART within eight weeks while all TB‐free contacts did so within 48 hours of the outreach intervention. Although evidence supports same‐day ART initiation, some studies have demonstrated patient unreadiness or insufficient time to rule‐out OIs prior to rapid ART initiation [43, 52, 53, 54, 55, 56, 57]. Our experience shows that patients’ counselling combined with use of POC‐based OI‐testing is feasible for rapid ART initiation.

Fourteen percent of the study population failed to attend their six‐month visit within a 30‐day window. Previous studies in Mozambique with less stringent definitions reported higher LTFU proportions [58, 59, 60].

Overall mortality in TB‐index cases was 21%, which is in line with the results of previous studies in the district [61, 62]. The vast majority of these deaths occurred among these TB/HIV cases with AHD during hospital admission. The recommendations for AHD management may need to be tailored to the inpatient population in order to further reduce its high mortality. By contrast, six‐month mortality among the 30 contacts starting ART was zero. This is particularly relevant considering that 29% met criteria for AHD at enrolment. Several reasons may account for this. First, the contacts with TB were effectively identified. Detecting TB and cryptococcal infection before ART initiation is critical to avoid immune reconstitution inflammatory syndrome (IRIS), a major cause of early post‐ART initiation mortality [5, 8, 9, 11, 25, 35, 63]. Of note, CrAg and TB‐LAM study indications were broader than those recommended at the time and performed among all patients with <200 cells/μL (regardless of symptoms) to increase operationalization [4, 21, 64, 65, 66, 67, 68, 69, 70]. Finally, all but two contacts received IPT, a life‐saving intervention that is still very low implemented [25, 32, 71, 72, 73].

The only independent predictor of attrition found was male sex. We also found a moderate evidence in favour of an association between anaemia, a known predictor of mortality in TB/HIV patients, and a two‐fold increase in the hazard of attrition, likely limited by the sample size [74, 75, 76]. TB‐LAM positivity and enrolment due to poor ART adherence were also significantly associated with attrition in the univariable analysis, both having been reported earlier [19, 35, 68, 77, 78, 79]. The latter is a reminder of the alarming fact that today, ART‐experienced individuals account for 40% to 60% of AHD cases presenting to care in SSA [1, 37, 65]. The small sample size warrants a prudent interpretation of these findings.

This study has some limitations. First, integrating our study within an existing community‐based TB‐contact intervention limits its replicability to similar conditions and may overestimate AHD prevalence, due to the high TB burden of the target population (TB index and contacts). Second, the community intervention would have greatly benefited from POC strip tests discriminating above or below 200 CD4 cells/μL on finger‐prick samples [80, 81]. This would have allowed for off‐clinic CD4 count testing, enabling a full home‐based POC strategy [19, 32]. Also, for those on ART a HIV‐VL POC‐assay should have followed in order to detect virological failure, beyond targeting those with suboptimal adherence. [82, 83]. The lack of POC‐CD4 and POC‐VL assays in our intervention made it less sensitive and less specific to identify a subgroup of HIV‐positive individuals that could have greatly benefitted from our intervention. Moreover, under routine programmatic conditions, participants that were clear suspects for ART failure (ART‐experienced individuals with TB and/or low CD4 counts) did not benefit from an ART switch during the 6‐month follow‐up period. WHO recommends expedited ART switch for AHD patients but this has not consistently translated into national recommendations, rather, patients with AHD usually endure the same long process of the general HIV‐positive population consisting of VL determinations repeated every three months, which is challenging in districts with non‐existent VL‐testing capacity [4, 28, 84, 85, 86]. The rollout of multi‐disease testing platforms such as GeneXpert which has POC‐VL capacity will increase the operationality of the recommended “package of care” for ART‐experienced patients with AHD [87, 88]. Third, we limited our ancillary intervention to adults because the TB‐contact tracing intervention (Xpatial‐TB) was already providing children with a provision of care far exceeding the package recommended by the WHO for AHD. Also, due to the limited sample size, we could only estimate risk‐factors of attrition in TB‐index cases. Finally, our small study sample limited our capacity to draw statistically significant conclusions.

Our study has also several strengths. To our knowledge, it is the first study attempting to use TB‐ACF to find AHD cases in the community. The WHO‐recommended provision of care was embedded within routine programmatic conditions in a high HIV and TB burden area. Also, it provides the first published estimates of CrAg prevalence among TB/HIV patients in Mozambique. Moreover, TB diagnosis in HIV‐positive TB contacts was based on novel assays including Ultra^®^ and TB‐LAM, not always routinely available. Finally, this is the first experience that we know reporting the capacity to achieve same‐day ART initiation in newly HIV‐diagnosed TB contacts reached in their communities.

## 5 Conclusions

This study unveiled a high burden of AHD, TB and cryptococcosis in a rural district of Mozambique and showed that TB contact‐tracing in communities offers an opportunity for TB‐HIV programmes to enhance detection and survival of HIV patients by identifying AHD cases and providing them with the WHO‐recommended package of care. Same‐day ART initiation was possible and safe in HIV‐positive TB‐free contacts with zero deaths among them. This POC‐based intervention exemplifies that comprehensive care provision is possible for patients with AHD in rural settings. If taken to scale, this strategy could contribute to reducing the still unacceptably high AIDS‐mortality in sub‐Saharan Africa.

## Ethics

This study was approved by the Institutional Review Board at CISM and the National Ethics Committee of Mozambique. The United States Centre for Disease Control and Prevention (CDC) received a determination of “non‐engaged in human subjects research” prior to the study. All participants provided voluntary written informed consent before enrolment.

## Competing interests

The authors declare no conflict of interest.

## Authors’ contributions

All authors contributed to the content and preparation of the manuscript and approved the final draft. Specific contributions: SI drafted the manuscript, and performed a literature review; SI, AM, AJ, GG, NC, HC, BS, JG and AB contributed to data collection and data analysis; AF and AC performed the statistical analysis. TC, TN, AG and EL provided expert opinion and revision of the manuscript for important content.
